# Construction of Sulfhydryl-Amino UiO-66/PVDF Membranes via Morphology Regulation for the Selective Separation of Artesunate

**DOI:** 10.3390/molecules31111885

**Published:** 2026-06-01

**Authors:** Kunyi Li, Ziyang Wang, Lingna Meng, Minjia Meng

**Affiliations:** School of Chemistry and Chemical Engineering, Jiangsu University, Zhenjiang 212013, China

**Keywords:** artesunate, coagulation bath, delayed-phase-inversion, structural analogues, hydrophobic and hydrophilic

## Abstract

Artesunate (ARU), a key derivative of artemisinin (ART), exhibits excellent water solubility and antimalarial activity due to its incorporation of a succinic acid group. However, the synthesis process of ARU often leaves behind ART with a highly similar structure and properties, making traditional separation methods ineffective for efficient separation. Developing selective separation technologies holds significant importance. Based on previous studies, in work involving the preparation of bidentate MOFs with different ligands, bidentate MOFs containing thiol/amino groups have been found to exhibit outstanding adsorption capacity and selectivity for ARU molecules. Among these, -NH_2_ forms hydrogen bonds with -COOH in ARU, while -SH interacts non-specifically with Aru, significantly enhancing the adsorption effect. This study employed a delayed inversion method to prepare a sulfhydryl-amino UiO-66/PVDF hybrid membrane (UiO-66-SH/NH_2_/PVDF) by adjusting the composition of the coagulation bath, which was used for efficient separation of ART/ARU. The effects of ethanol ratio in the coagulation bath on membrane structure and performance were systematically investigated. Results showed that increasing the ethanol ratio delays phase transition, promotes MOF material enrichment on membrane pore surfaces, and forms more abundant pore structures. When the ethanol-to-water volume ratio was 1:1, the UiO-66-SH/NH_2_/PVDF membrane exhibited optimal pore structure and highest water flux. Static permeation experiments demonstrated that the membrane achieved effective separation of ARU and ART for 8 h, maintaining stable selective adsorption performance after five cycles. This study reveals the critical role of morphology regulation in separating structural analogs, providing new materials and theoretical foundations for efficient separation of artemisinin-based compounds.

## 1. Introduction

Artemisinin (ART) [1,2,3,4], a sesquiterpene lactone featuring an endoperoxide bridge, serves as the first-line therapeutic agent for cerebral and falciparum malaria due to its high efficacy and favorable safety profile. Artesunate (ARU) [2,5], a semisynthetic derivative of ART obtained via esterification, exhibits not only markedly improved water solubility and antimalarial potency but also cytotoxic effects against various human cancers—including leukemia, colon cancer, and melanoma—suggesting its potential as a novel anticancer agent [6,7,8]. The therapeutic efficacy of ARU is closely associated with its purity; however, residual ART, which shares highly similar structural and physicochemical properties with ARU, often persists during synthesis. Conventional separation methods lack sufficient selectivity to achieve efficient resolution of these two compounds [9,10,11]. Consequently, the development of advanced materials and technologies enabling precise ART/ARU separation is of considerable importance.

Metal–organic frameworks (MOFs) [12,13,14,15,16,17], a class of porous hybrid materials formed via the self-assembly of metal ions/clusters with organic ligands under mild conditions [18], have demonstrated substantial promise in separation applications through ligand-functionalized modulation of host–guest interactions [19,20,21]. In our previous work, we developed UiO-66-NH_2_ for ART/ARU separation, which outperformed conventional molecularly imprinted materials in both ARU adsorption capacity (approximately 60 mg g^−1^) and selectivity factor (around 40) [11]. Nevertheless, this adsorption capacity remains suboptimal for porous MOFs. Given the pronounced hydrophobicity of both ART and ARU, the selective adsorption behavior of hydrophilic UiO-66-NH_2_ primarily relies on hydrogen bonding between surface amino groups and the carboxyl moiety of ARU. Most hydrophobic molecules are excluded from the MOF channels due to hydrophobic repulsion, thereby limiting adsorption capacity [22,23]. To address this limitation, we propose introducing controlled hydrophobic sites while preserving the overall hydrophilicity of the MOF matrix. This strategy is expected to enhance ARU adsorption while suppressing nonspecific ART binding.

Building upon this rationale, we previously synthesized 2,5-dimercaptopropanedioic acid and 2-aminopyromellitic acid as dual ligands to construct a mercapto-functionalized UiO-66-NH_2_ framework, which significantly enhanced ARU adsorption capacity. To facilitate practical applications, the present study develops MOF-PVDF composites via a delayed phase inversion method [24,25] to overcome challenges associated with powder recovery and discontinuous operation in MOF-based processes [26,27]. Notably, membrane separation performance is predominantly governed by morphological characteristics, with the composition of the coagulation bath during phase inversion playing a critical role in regulating pore architecture and surface properties [28]. Current understanding of how coagulation bath conditions influence the microstructure and separation efficiency of MOF/PVDF composites remains limited. In particular, the separation mechanisms governing structurally analogous compounds such as ART and ARU warrant further investigation.

Accordingly, this study systematically investigates the effects of ethanol concentration in the coagulation bath on the morphology, pore structure, and surface properties of a UiO-66/PVDF hybrid membrane (UiO-66-SH/NH_2_/PVDF) constructed via sulfhydryl/amino dual functionalization. By exploring the intrinsic relationships among phase transition kinetics, MOF migration behavior, pore formation, and hydrophilic/hydrophobic balance, we aim to elucidate the mechanism by which membrane morphology regulation influences separation selectivity for ART/ARU. This work provides a new theoretical and material foundation for achieving efficient membrane-based separation of artemisinin derivatives.

## 2. Results and Discussion

### 2.1. Characterization

#### 2.1.1. SEM

**Figure 1:**
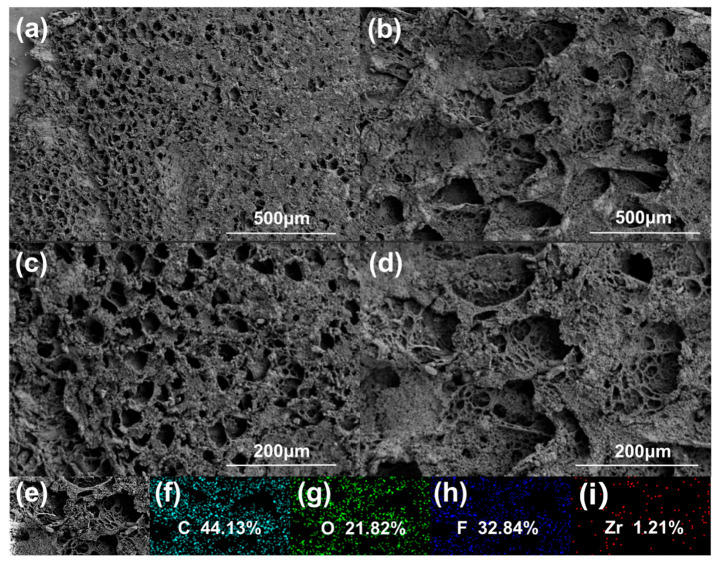
The SEM images of optimally prepared UiO-66-SH/NH_2_/PVDF membrane 1:1 (**a**,**c**), 1:5 (**b**,**d**), mapping and elements distribution images of 1:1 (**e**–**i**).

Figure 1 displays the scanning electron microscopy (SEM) images of the UiO-66-SH/NH_2_/PVDF membrane (1:1 and 1:5) membranes. As observed from the figures, compared to the 1:5 membrane, the 1:1 UiO-66-SH/NH_2_/PVDF membrane exhibited a more abundant and uniformly distributed pore structure, indicating superior pore-forming effectiveness. This difference may be attributed to variations in the composition of the coagulation bath, which led to differences in the rate and efficiency of solvent exchange from the cast film, as well as differences in the rate and extent of outward migration of the hydrophilic MOF (UiO-66-SH/NH_2_) from the membrane matrix. Furthermore, elemental mapping analysis revealed the homogeneous distribution of C, F, O, and Zr elements across the membrane surface. The respective atomic percentages of these elements were 44.13%, 32.84%, 21.82%, and 1.21%.

#### 2.1.2. Membrane Flux Assay and Contact Angle Test

**Figure 2:**
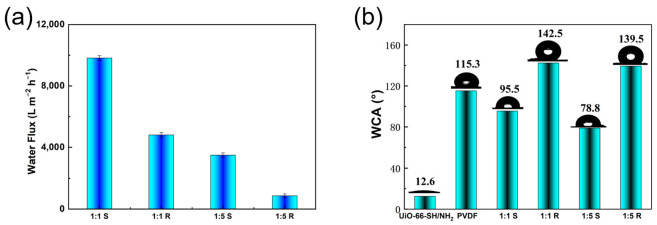
The water flux of optimally prepared UiO-66-SH/NH_2_/PVDF membranes (**a**) and WCA data of optimally prepared UiO-66-SH/NH_2_/PVDF membranes (**b**).

As shown in Figure 2, the water flux and water contact angle (WCA) data of the UiO-66-SH/NH_2_/PVDF membrane were presented. From Figure 2a, it was observed that the UiO-66-SH/NH_2_/PVDF membrane (1:1) exhibited significantly higher water flux than of the membrane (1:5), reaching up to 9816 L m^−2^ h^−1^. This superior performance was likely attributed to the more abundant and uniformly distributed pore structure of the UiO-66-SH/NH_2_/PVDF membrane (1:1). Additionally, a noticeable difference in flux was observed between the front and back sides of the membrane, with the front side showing much higher permeability. This asymmetry was possibly caused by the low-temperature treatment of the glass plate prior to phase inversion, which led to variations in pore size distribution across the membrane cross-section.

As shown in Figure 2b, the water contact angle (WCA) of the modified UiO-66-SH/NH_2_/PVDF membranes differed markedly from that of the pure PVDF membrane. Notably, the 1:1 membrane exhibited a higher WCA compared to the 1:5 membrane, indicating enhanced hydrophobicity with increased MOF loading. This trend can be attributed to the synergistic effect of surface chemistry and morphology induced by the high content of UiO-66-SH/NH_2_. Although the MOF particles contain hydrophilic amino groups (-NH_2_), they were also functionalized with hydrophobic thiol groups (-SH). At the high loading of 1:1, two key factors come into play: first, the abundant MOF particles tend to migrate toward the membrane surface during phase inversion, enriching the surface with thiol groups. These hydrophobic -SH groups lower the surface energy and contribute to increased water repellency. Second, the high MOF content promotes the formation of a rich and uniformly distributed porous structure, as observed in the 1:1 membrane. This morphology significantly increases surface roughness, creating an uneven topography that enhances the hydrophobic effect by trapping air at the solid–liquid interface. Consequently, the combination of surface enrichment of hydrophobic thiol groups and increased surface roughness from the porous architecture outweighs the hydrophilic contribution of the amino groups, resulting in a higher water contact angle for the 1:1 membrane.

#### 2.1.3. Fourier Transform Infrared (FT-IR) Spectra and X-Ray Diffraction Patterns

**Figure 3:**
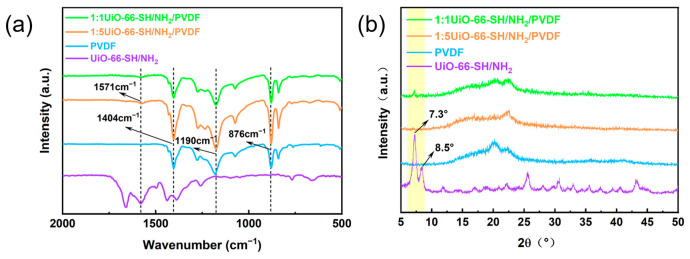
X-ray diffraction patterns of UiO-66-SH/NH_2_/PVDF membrane (1:1 and 1:5) and PVDF (**a**), FT-IR spectra of UiO-66-SH/NH_2_/PVDF membrane (1:1 and 1:5) and PVDF (**b**).

Figure 3a showed the Fourier transform infrared (FT-IR) spectra of the 1:1 and 1:5 UiO-66-SH/NH_2_/PVDF membranes. The absorption peak observed at 876 cm^−1^ was attributed to the characteristic crystalline phase variation in PVDF. The peak at 1057 cm^−1^ corresponded to the out-of-plane bending vibration of C-H bonds. The absorption at 1190 cm^−1^ originated from the stretching vibration of the C-F bond in PVDF, while the peak at 1404 cm^−1^ was caused by the vibration of CH_2_ groups in the PVDF polymer units. Notably, the absorption peak at 1571 cm^−1^ was assigned to the stretching vibration of the C=O bond in the organic ligand of UiO-66-NH_2_ [29]. These characteristic peaks collectively confirmed the successful preparation of both the 1:1 and 1:5 UiO-66-SH/NH_2_/PVDF membrane materials.

The crystal structures of thiol-functionalized UiO-66-SH/NH_2_ and UiO-66-SH/NH_2_/PVDF were analyzed using X-ray diffraction, as shown in Figure 3b. The characteristic diffraction peaks of UiO-66-SH/NH_2_ at 2θ = 7.3° and 8.5° are assigned to the (1 1 1) and (0 0 2) crystal planes [30], respectively. A distinct characteristic diffraction peak of UiO-66-SH/NH_2_ is observed at 2θ = 7.3° in UiO-66-SH/NH_2_/PVDF, indicating that the thiol-functionalized UiO-66-SH/NH_2_ has been successfully loaded onto the membrane surface while maintaining the integrity of its crystal structure. Additionally, the migration amount of MOF to the membrane surface in the 1:1 UiO-66-SH/NH_2_/PVDF is greater than that in the 1:5 UiO-66-SH/NH_2_/PVDF.

#### 2.1.4. Aperture Distribution

**Figure 4:**
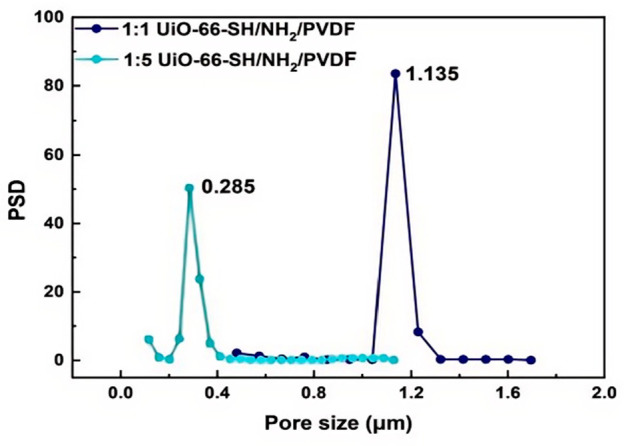
The pore size distribution of UiO-66-SH/NH_2_/PVDF membrane (1:1 and 1:5).

The pore size of the membrane significantly influenced its adsorption performance, particularly when active adsorption sites were present on the pore surfaces [31]. Pore dimensions affected the distribution of these active sites, thereby further modulating the adsorption behavior of the membrane material [32]. Figure 4 presented the pore size distribution data for the 1:1 and 1:5 UiO-66-SH/NH_2_/PVDF membrane. It was observed that the 1:1 membrane exhibited a larger average pore size (1.135 μm), whereas the 1:5 membrane showed a considerably smaller pore size (0.285 μm). This discrepancy was likely due to differences in the composition of the coagulation bath during the phase inversion process, which affected the rate and extent of outward migration of hydrophilic UiO-66-SH/NH_2_ particles from the membrane matrix. These variations in migration behavior led to differences in pore size and distribution, ultimately influencing the adsorption performance of the membranes.

### 2.2. Performance Evaluation

#### 2.2.1. Adsorption Isotherm

**Figure 5:**
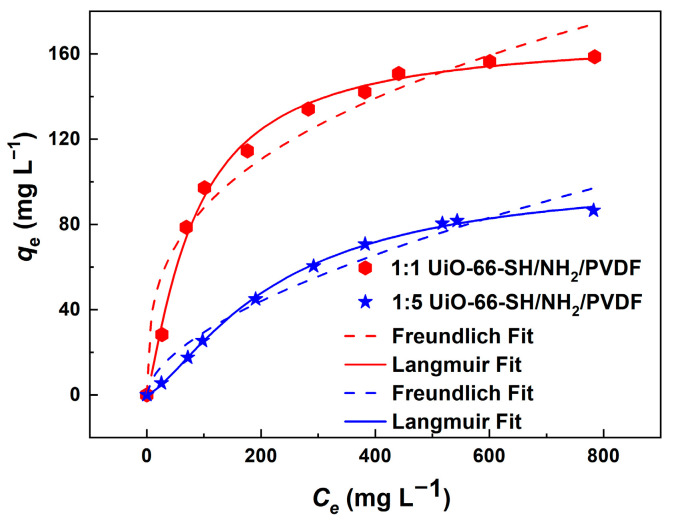
The adsorption isotherm fitting toward ARU of UiO-66-SH/NH_2_/PVDF membrane (1:1 and 1:5).

To evaluate the adsorption isotherm performance of the 1:1 and 1:5 UiO-66-SH/NH_2_/PVDF membrane for the target substance (ARU), isothermal adsorption tests were conducted, and the experimental data were nonlinearly fitted using both the Freundlich and Langmuir models. The better-fitting model was determined by comparing the correlation coefficients (*R*^2^) of the fitting results. Figure 5 showed the equilibrium adsorption capacities of the two membranes in ARU solutions with gradient concentrations. It was observed that both membranes exhibited similar trends in ARU adsorption. With increasing ARU concentration, the equilibrium adsorption capacity of both the 1:1 and 1:5 membranes exhibited a relatively rapid increase during the initial stage. Furthermore, the 1:1 membrane demonstrated a higher adsorption capacity for ARU compared to the 1:5 membrane. Table 1 summarizes the parameters obtained from the Freundlich and Langmuir model fitting. Both graphical analysis and correlation coefficients indicated that the Langmuir model provided a better description of the adsorption behavior, suggesting monolayer adsorption where intermolecular interactions were negligible. Based on the Langmuir model, the maximum adsorption capacities were calculated to be 168.59 mg g^−1^ for the 1:1 membrane and 103.86 mg g^−1^ for the 1:5 membrane, with *R*^2^ values of 0.9902 and 0.9983, respectively. In contrast, the Freundlich model yielded lower *R*^2^ values of 0.9439 (1:1) and 0.9619 (1:5). Additionally, the Freundlich coefficients n were greater than 1 (3.0082 and 1.7223, respectively), indicating a favorable adsorption tendency of both membranes toward ARU molecules.

#### 2.2.2. Adsorption Kinetics

**Figure 6:**
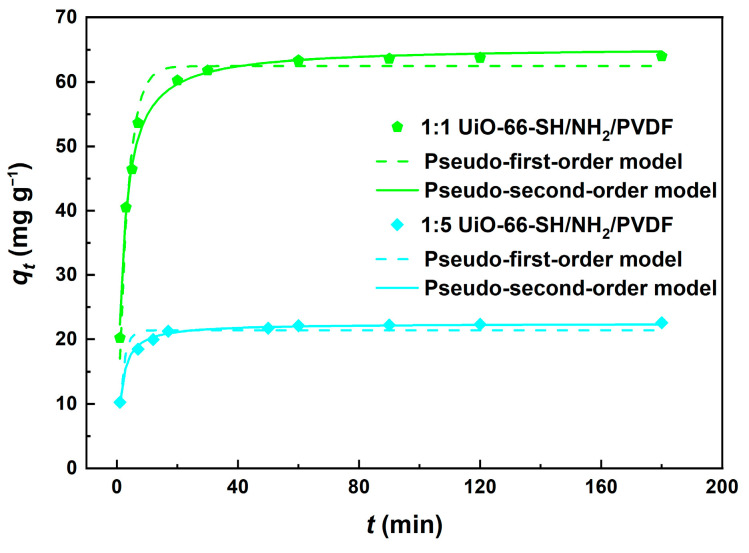
The kinetic model fitting of over ARU of UiO-66-SH/NH_2_/PVDF membrane (1:1 and 1:5).

To investigate the adsorption kinetics of the two membrane materials toward target molecules, dynamic adsorption tests were conducted with sampling at specific time intervals to calculate adsorption capacities. The results are presented in Figure 6. As observed, both membranes reached adsorption equilibrium at approximately 60 min during the entire adsorption process. In terms of adsorption performance, the 1:1 UiO-66-SH/NH_2_/PVDF membrane exhibited a higher adsorption capacity than of the 1:5 membrane. This was likely attributed to the delayed phase inversion effect during the synthesis of the 1:1 membrane, which promoted greater migration of active adsorption components (MOF) to the membrane pore and surfaces of the membrane. As a result, these components interacted more effectively and thoroughly with the target molecules (ARU) during adsorption, leading to enhanced adsorption capacity.

In the initial stage of adsorption, the relatively abundant active sites enabled rapid binding with target molecules, causing a sharp increase in adsorption capacity. As the process continued, partial occupation of the active sites by adsorbed molecules led to a gradual slowdown in the binding rate. When the active sites approached saturation, the increase in adsorption capacity significantly diminished and eventually stabilized, reaching adsorption equilibrium.

From a kinetic modeling perspective, the experimental data were fitted using two common adsorption kinetic models: pseudo-first-order and pseudo-second-order. The corresponding equations can be found in Section 2.4 and Section 2.5.

The fitted parameters for both membrane materials are summarized in Table 2. It was observed that for both the 1:1 and 1:5 membrane, the correlation coefficient (*R*^2^) of the pseudo-second-order model was significantly higher than that of the pseudo-first-order model (0.9924 > 0.9751 for 1:1, 0.9923 > 0.8811 for 1:5). Moreover, the calculated equilibrium adsorption capacity (*q_e, cal_*) derived from the pseudo-second-order model was closer to the experimentally measured value (*q_e, exp_*) compared to that from the pseudo-first-order model. These results indicate that the pseudo-second-order model more accurately describes the adsorption kinetics of both membranes for the target molecules, suggesting that the adsorption process is primarily governed by chemical adsorption mechanisms.

#### 2.2.3. Adsorption Selectivity

**Figure 7:**
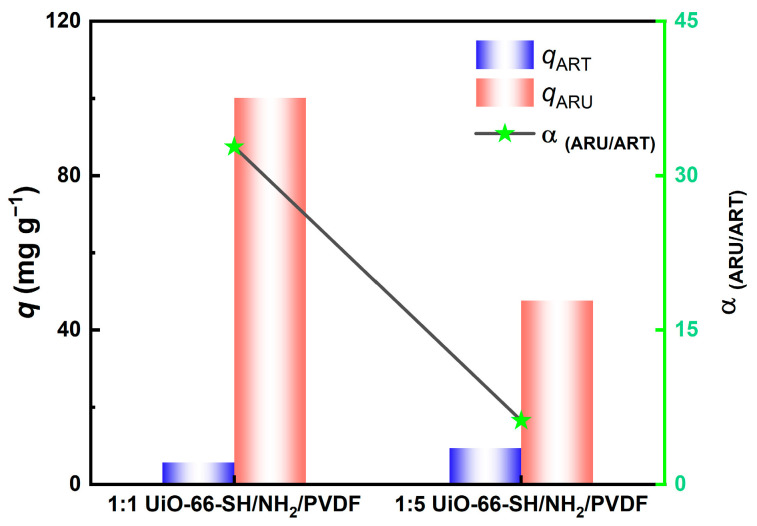
The selective property on ARU/ART of 1:1 and 1:5 UiO-66-SH/NH_2_/PVDF.

Figure 7 shows the adsorption selectivity of 1:1 type S1-UiO-66-NH_2_/PVDF membrane material and 1:5 type S1-UiO-66-NH_2_/PVDF membrane material for ARU/ART. From Figure 7, it can be seen that the 1:1 type exhibits a more prominent adsorption selectivity for ARU, with a selectivity coefficient α reaching 32.791, which is significantly higher than that of the 1:5 type (*α* = 6.200). Table 3 is a comparison of our research with other studies that include separation factors and adsorption capacities as reported results.

#### 2.2.4. Static Permeation Performance

**Figure 8:**
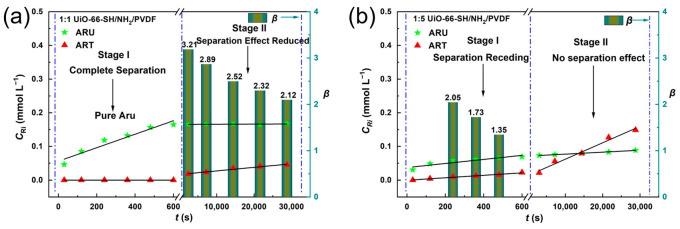
The static permeation diagram of 1:1 UiO-66-SH/NH_2_/PVDF membrane (**a**) and the static permeation diagram of 1:5 UiO-66-SH/NH_2_/PVDF membrane (**b**).

To evaluate the static permeation performance of the 1:1 and 1:5 UiO-66-SH/NH_2_/PVDF membranes, a 2.0 mmol L^−1^ ARU ethanol solution was used as the receiving phase and pure ethanol as the feed solution. The results were shown in Figure 8.

Figure 8a presented the permeation data of the 1:1 membrane, where the entire process could be divided into two distinct stages. In the first phase (0–10 min), nearly only ARU passed through, with no ART, successfully extending this time period compared to previous studies. This enhancement was attributed to the optimized 1:1 membrane structure, which featured larger and more uniform pores with a higher density of active UiO-66-SH/NH_2_ adsorption sites within the pore channels. As permeation continued into the second stage (after 10 min), the gradual occupation of these active sites led to decreased selective permeability for ARU, allowing smaller ART molecules to diffuse through the membrane into the permeation phase.

Figure 8b showed the permeation performance of the 1:5 membrane. During the initial stage (0–2 min), only ARU passed through, though the concentration of ARU in the permeate was lower than that of the 1:1 membrane. As permeation continued (2–10 min), the separation efficiency progressively declined, accompanied by a gradual decrease in the separation factor (*β*). In the subsequent stage (after 10 min), the limited active adsorption sites became saturated, resulting in a rapid increase in ART concentration and complete loss of ARU separation capability.

### 2.3. Recyclability

**Figure 9:**
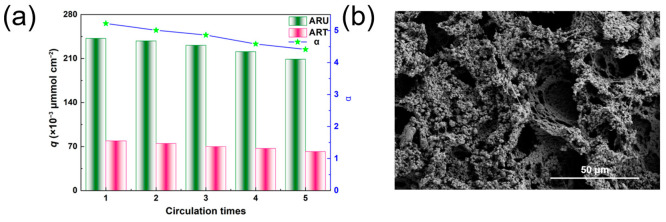
The regeneration-recycling performance of 1:1 UiO-66-SH/NH_2_/PVDF membrane (**a**) and the SEM scan of the 1:1 UiO-66-SH/NH_2_/PVDF membrane after the fifth cycle (**b**).

Recyclability represented a crucial indicator for assessing the performance of adsorption materials. The adsorbed membrane material was regenerated through elution with a formic acid–ethanol mixed solution, followed by washing and drying. Adsorption experiments were then repeated under identical conditions to evaluate changes in adsorption capacity and selectivity. Figure 9a illustrated the variation in adsorption capacity and selectivity coefficient of the 1:1 UiO-66-SH/NH_2_/PVDF membrane over five consecutive recycling experiments. As shown in the figure, after five cycles, the adsorption capacity of the membrane decreased by only 13.7%, while the selectivity separation coefficient *α* decreased by merely 15.6%. Figure 9b illustrated the SEM scan of the 1:1 UiO-66-SH/NH_2_/PVDF membrane after the fifth cycle, it can be seen that the membrane morphology remains good after the fifth cycle. These results demonstrated that the 1:1 UiO-66-SH/NH_2_/PVDF membrane possessed excellent regeneration capability and cycling stability.

### 2.4. Materials and Methods

#### 2.4.1. Materials

Tetraclorozirconium (ZrCl_4_, 98%), 2,5-Dimercaptoterephthalic acid (C_8_H_6_O_4_S_2_, DMCA, 98%), 2-Aminophenylbenzoic acid (C_8_H_7_NO_4_, ATA, 98%),, anhydrous ethanol (C_2_H_4_O_2_, AcOH, 99.5%/HPLC), acetic acid (HAc, 99.5%), acetonitrile (MeCN, 99%), methanol (MeOH, 98%), dimethylsulfoxide (C_2_H_6_OS, DMSO, 98%) were supplied by Sinopharm Chemical Reagent Co., Ltd. (Shanghai, China), N,N-Dimethylformamide (HCON(CH_3_)_2_, DMF, 98%), artemether (C_15_H_24_O_5_, ARU, 99%) were purchased from Aladdin Biochemical Technology Co., Ltd. (Shanghai, China), artemisinin (C_12_H_22_O_5_, ART, 99%) polyvinylidene chloride ((CH_2_CF_2_)_n_, PVDF, Mw = 110,000) was supplied by Merck Millipore China Co., Ltd. (Shanghai, China).

#### 2.4.2. Preparation of UiO-66-SH/NH_2_

The synthesis method of UiO-66-SH/NH_2_/PVDF was improved based on the existing method. The specific steps are as follows: 0.2330 g (1.0 mmol) of ZrCl_4_ metal precursor, 0.1630 g (0.9 mmol) of ATA, 0.0230 g (0.1 mmol) of DMCA were weighed separately and placed in a 50 mL of polytetrafluoroethylene inner container. Then, 3.6 mL of HAc and 30 mL of DMF were added. After ultrasonic treatment for 10 min, it was stirred at room temperature for 1 h. The reaction was carried out at 120 °C for 24 h. After cooling to room temperature, it was washed alternately with deionized water and ethanol. It was then vacuum-dried at 60 °C for 12 h. After grinding, methanol was added for activation. The volume of methanol was in the ratio of *v*:*m* = 50 mL:0.1 g, and activation time was 24 h. During the activation process, methanol needed to be replaced at regular intervals. After the activation is completed, it should be dried under vacuum at 80 °C for 12 h, then grounded to obtain UiO-66-SH/NH_2_ as a reserve.

#### 2.4.3. Preparation of the Sulfhydryl-Amino UiO-66/PVDF Membrane (UiO-66-SH/NH_2_/PVDF)

2.1 g of PVDF powder was dissolved in 22.9 g of DMSO. After ultrasonic treatment for 2 min, it was placed in a water bath at 50 °C and stirred for 2 h. Subsequently, 0.1 g of UiO-66-SH/NH_2_ was dissolved in 5 g of DMSO under ultrasonic dispersion for 2 min. The mixture was then added to the above solution and stirred for 1 h to form the casting solution. After the reaction was completed, the casting solution was evenly spread onto the surface of a pre-cooled glass plate. The plate was immediately immersed in a coagulation bath consisting of a mixture of ethanol and deionized water (with volume ratios ranging from 1:1 to 1:5) for 2 h. Subsequently, the membrane was transferred to a second coagulation bath containing pure deionized water and soaked for another 24 h, during which the deionized water was replaced every 8 h. After complete removal of the DMSO, the membrane was dried under vacuum to obtain the UiO-66-SH/NH_2_/PVDF membrane with a non-uniform pore size optimized via the phase inversion method. The resulting membrane could be cut into the desired shape and stored in filter paper or weighing paper for future use.

#### 2.4.4. Membrane Flux Assay and Water Contact Angle Test

During the flux test, the membrane material was fixed in the self-made flux testing instrument, and a certain volume of deionized water was added. Under a pressure of 0.1 Mpa, the liquid continuously passed through the membrane material. The effective area of the membrane was 3.4 cm^2^. The flux *J* (L m^−2^ h^−1^) of the membrane could be calculated using the Formula (1) [35]:

(1) $$J=\frac{V}{st}$$

where *V* (L) is the permeate volume, *s* (m^2^) is the effective membrane area, and *t* (h) is the filtration time.

The flux of the membrane was measured multiple times to ensure data reliability. For the contact angle analysis, the static water contact angle was measured using an optical surface analyzer to evaluate the surface hydrophilicity/hydrophobicity of the membrane.

#### 2.4.5. Kinetic Adsorption Test

First, an ethanol solution of ARU with a concentration of 200 mg L^−1^ was prepared as the stock solution. A predetermined mass of the membrane material was added to centrifuge tubes containing 10 mL of this stock solution. The tubes were then placed in a water bath shaker at 25 °C for adsorption under oscillation. Samples were collected at different time intervals (1, 3, 5, 7, 10, 30, 60, 90, 120, and 180 min), and their concentrations were determined using high-performance liquid chromatography (HPLC). The adsorption amount *q_t_* (mg g^−1^) at each time point was calculated using the following Formula (2) [36]:

(2) $$q_{t}=\frac{(C_{0}-C_{t})V}{M}$$

where *q_t_* (mg g^−1^) represents the adsorption capacity corresponding to a certain time point, *C*_0_ (mg L^−1^) is the initial concentration of ARU ethanol solution, *C_t_* (mg L^−1^) is the concentration of the sample taken at a certain time point, *V* (L) is the total volume of the solution, and *M* (g) is the mass of the membrane material used during the adsorption process.

### 2.5. Isotherm Adsorption Experiment

In order to study the isotherm adsorption characteristics of the UiO-66-SH/NH_2_/PVDF membrane, different concentrations of ARU ethanol solutions (100, 200, 300, 400, 500, 600 and 800 mg L^−1^) were prepared. A certain mass of UiO-66-SH/NH_2_/PVDF membrane material was added to 10 mL of different concentration ARU ethanol solutions in centrifuge tubes, and the tubes were placed in a water bath at 25 °C for 3 h of oscillation for adsorption. The supernatant was filtered and the concentration was tested using high-performance liquid chromatography. The *q_e_* (mg g^−1^) can be calculated according to the following Formula (3):

(3) $$q_{e}=\frac{(C_{0}-C_{e})V}{M}$$

where *q_e_* (mg g^−1^) represents the adsorption capacity of the MOF when it reaches the adsorption equilibrium, *C*_0_ (mg L^−1^) is the initial concentration of the ARU ethanol solution, *C_e_* (mg L^−1^) is the equilibrium concentration of the solution after adsorption is completed, *V* (L) is the total volume of the solution, *M* (g) is the mass of the adsorbent used during the adsorption process.

### 2.6. Adsorption Selectivity

To test the adsorption selectivity of MOF materials for Aru and Art, competitive experiments were conducted. Specifically, a binary ethanol solution containing both Aru and Art at concentrations of 200 mg L^−1^ was prepared. After adsorption, the mixture was centrifuged, and the supernatant was filtered through a 0.22 μm organic membrane filter. The residual concentrations of ARU and ART in the supernatant were then determined using HPLC. The HPLC mobile phase consisted of high-performance liquid chromatography-grade acetonitrile and pH 4.5 acetic acid aqueous solution (volume ratio *v*:*v* = 60:40), with a flow rate of 1 mL min^−1^, column temperature of 30 °C, and detection wavelength of 213 nm. The competitive distribution coefficient *K_d_* (mL g^−1^) of MOF for Aru and Art was calculated using Formula (4), and the selectivity coefficient α was calculated using Formula (5).

(4) $$K_{d}=\frac{q_{e}}{C_{e}}$$

where *q_e_* (mg g^−1^) represents the adsorption amount of ARU/ART when the adsorption equilibrium is reached. *C_e_* (mg L^−1^) represents the residual concentration of ARU/ART in the solution when adsorption equilibrium is reached.

(5) $$\alpha =\frac{K_{d(\mathrm{Aru})}}{K_{d(\mathrm{Art})}}$$

where *K_d_*_(*Aru*)_ refers to the distribution coefficient for ARU in the mixed solution, and *K_d_*_(*Art*)_ refers to that for ART.

### 2.7. Static Permeation Experiment

In order to test the selective permeation performance of the optimized UiO-66-SH/NH_2_/PVDF membrane, a static permeation experiment with ARU/ART binary component as the target substance was also conducted. The permeation experiment was carried out in the H tube, with the UiO-66-SH/NH_2_/PVDF membrane placed in the middle of the two chambers of the H tube (the effective permeation area was 27.5 cm^2^), and it was fixed with adhesive tape and cling film. Due to the structural differences between the front and back sides of the UiO-66-SH/NH_2_/PVDF membrane, the side with larger pore size was facing the liquid phase, and the side with smaller pore size was facing the permeation phase, allowing the liquid to stay in the membrane pores for as long as possible, thereby improving the adsorption and separation effect. The feed solution was a binary mixture of 0.6 mmol of ARU and ART, and the receiving phase was an ethanol solution, both with a volume of 150 mL. At room temperature, magnetic stirring was performed, and samples were taken at multiple time points (1, 3, 5, 7, 10, 30, 60, 90, 120, 240, 360 and 480 min) and their concentrations were tested using high-performance liquid chromatography. The adsorption amount *q_t_* (mmol L m^−2^) and the selectivity coefficient α could be calculated using the previous Formulas (3)–(5).

## 3. Conclusions

This study developed a sulfhydryl/amino dual-functionalized UiO-66-NH_2_@PVDF membrane via delayed phase inversion to address the challenges of separating artemisinin (ART) and artesunate (ARU). This approach systematically investigated the influence of coagulation bath composition on membrane structure and separation performance. The results demonstrated that adjusting the volume ratio of ethanol to deionized water in the coagulation bath effectively modulated the phase inversion rate and the migration behavior of the hydrophilic UiO-66-SH/NH_2_ toward the membrane pore channel surfaces, thereby optimizing the pore size distribution. The 1:1 membrane exhibited enhanced hydrophobicity due to synergistic surface enrichment of thiol groups and increased roughness from porous architecture. The adsorption followed pseudo-second-order kinetics and Langmuir isotherm models. Static permeation achieved complete ART/ARU separation within 10 min, with significantly increased ARU concentration in the permeate. In summary, this study provides a new theoretical foundation and material basis for the application of MOF-based mixed matrix membranes in the separation of structural analogs and offers a feasible pathway for the development of membrane separation technology for artemisinin-derived compounds.

## Figures and Tables

**Table 1:** The isotherm models fitting parameters of UiO-66-SH/NH_2_/PVDF membrane (1:1 and 1:5).

Model	Parameter	1:1 UiO-66-SH/NH_2_/PVDF	1:5 UiO-66-SH/NH_2_/PVDF
Langmuir model	*R* ^2^	0.9902	0.9983
	*K_L_* (L mg^−1^)	0.0047	0.0005
	*q_m, cal_* (mg g^−1^)	168.5923	103.8625
Freundlich model	*R* ^2^	0.9439	0.9619
	*K_F_* (mg g^−1^)	18.9894	2.2065
	*n*	3.0082	1.7223

**Table 2:** The kinetic model fitting parameters of two membranes over ARU.

Model	Parameter	1:1 UiO-66-SH/NH_2_/PVDF	1:5 UiO-66-SH/NH_2_/PVDF
Pseudo-first-order model	*q_e, exp_* (mg g^−1^)	64.0137	22.5562
	*q_exp, cal_* (mg g^−1^)	62.4679	21.3924
	*k*_1_ (min^−1^)	0.3168	0.6097
	*R* ^2^	0.9751	0.8811
Pseudo-second-order model	*q_exp, cal_* (mg g^−1^)	65.4612	22.4543
	*k*_2_ (g mg^−1^ min^−1^)	0.0079	0.0353
	*R* ^2^	0.9924	0.9923

**Table 3:** Comparison with results from other studies that include separation factors and adsorption capacities.

	Adsorption Capacity *q* (mg g^−1^)	Separation Factor *α*	Ref.
UiO-66-SH/NH_2_/PVDF	100.17	32.79157	This work
MIP@Ag@PDA@SiO_2_@PCM	18.89	2.03	[33]
SPIMs	45.89	3.0	[34]
GO/MXene based membranes	36.3	4.1	[10]

## Data Availability

The data are contained within the article.
